# Chemoproteomics approach to elucidating biosynthetic pathway of plant natural products

**DOI:** 10.3389/fpls.2024.1506569

**Published:** 2024-11-26

**Authors:** Quanyu Yin, Mengquan Yang

**Affiliations:** National Tobacco Cultivation, Physiology and Biochemistry Research Center, Flavors and Fragrance Engineering and Technology Research Center of Henan Province, College of Tobacco Science, Henan Agricultural University, Zhengzhou, Henan, China

**Keywords:** chemoproteomics, biosynthesis, natural product, affinity probe, medicinal plant

## 1 Introduction

**Figure 1:**
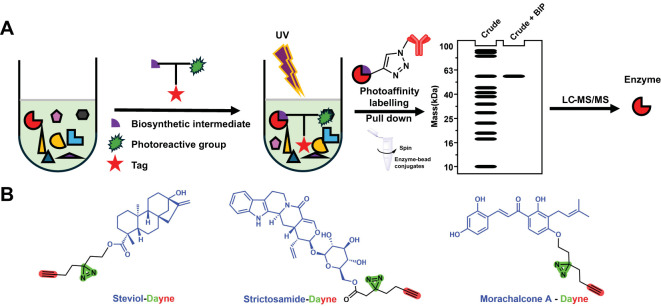
**(A)** Chemoproteomics approach to characterize the enzymes involved in the biosynthesis of plant natural products. **(B)** Reported photoaffinity-based probes for enzyme discovery.

Natural products derived from medicinal plants are a class of compounds with extensive biological activities, playing a crucial role in the pharmaceutical, food, and cosmetics industries. Due to their excellent physiological functions, increasing attention is being paid to the biosynthesis pathways of plant natural products (PNPs) (Kawatra et al., 2022; Halder and Jha, 2023). However, as market demand continues to grow, traditional harvesting and plant extraction methods exert immense pressure on the environment (Singh, 2023). In recent years, the rapid advancement of synthetic biology has offered new approaches for producing structurally complex bioactive small-molecule compounds using biotechnology (Hesami et al., 2023). Nevertheless, the lack of knowledge about biosynthetic pathways significantly impedes the large-scale biomanufacturing of natural products from medicinal plants. Unlike microorganisms, the biosynthetic genes for plant natural products are relatively dispersed across chromosomes, and medicinal plants often lack efficient genetic manipulation systems, which hinders the elucidation of their biosynthetic pathways. Recently, chemoproteomics based on activity probes has demonstrated great potential in elucidating plant natural product biosynthesis (steviol glycosides, camptothecin, chalcomoracin, etc.), as it enables the rapid identification of functional proteins interacting with substrates, thereby accelerating the discovery of biosynthetic pathways (Li et al., 2018; Zhou et al., 2018; Gao et al., 2020; Wong et al., 2020; Zhang et al., 2024) (**Figure 1**).

Traditional approaches have played a crucial role in advancing our understanding of plant natural product biosynthetic pathways, laying a foundation for emerging technologies like chemoproteomics. Gene knockout and RNA interference (RNAi) methods, for example, have been widely used to identify genes involved in biosynthetic pathways by observing phenotypic changes in metabolite production when specific genes are silenced (Zhao et al., 2016). Additionally, multi-omics approaches, such as transcriptomics, offer insights by gene coexpression, though these methods can be limited by the need for extensive data analysis and do not directly identify enzyme activities (Liu et al., 2024a; Swamidatta and Lichman, 2024). Heterologous gene expression, often in microbial and plant systems, has enabled functional analysis of individual genes or gene clusters by recreating biosynthetic pathways outside the native plant context (Lau and Sattely, 2015; Hong et al., 2022; Yang et al., 2024). However, traditional biochemical assays used to verify enzyme function may require large amounts of purified protein, a time-intensive process (Tatsis et al., 2017). These methods, while foundational, often fall short in dissecting complex pathways directly within plants, which is where chemoproteomics, with its activity-based probes and functional annotation capabilities, offers distinct advantages. Consequently, applying chemoproteomics technology to comprehensively analyze the biosynthesis of plant natural products not only has practical value for the rapid identification of functional genes involved in biosynthesis but also holds strategic significance for achieving large-scale production of medicinal plant natural products through synthetic biology (Zhang et al., 2022; Gao et al., 2023; Liu et al., 2023; Zhang et al., 2023; Golubova et al., 2024; Jiang et al., 2024; Liu et al., 2024b).

## 2 Workflow of affinity probes

**Figure 2:**
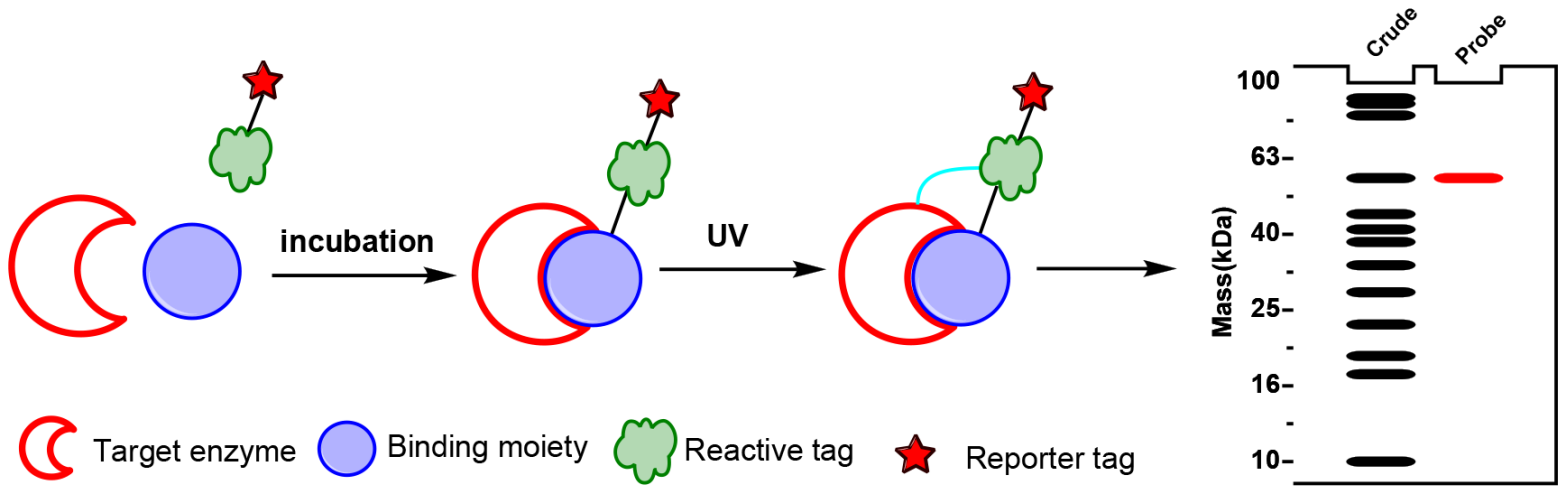
The workflow of affinity probes.

Affinity probes are specialized chemical tools used in chemoproteomics to isolate and identify active enzymes within complex biological samples, particularly those involved in plant natural product biosynthesis. These probes typically consist of a binding moiety that targets the enzyme’s active site, a reactive tag that enables enzyme capture through covalent attachment after activation and a reporter tag for detection. Effective affinity probe design requires specificity to mimic natural substrates, stability in biological conditions, and controlled reactivity to ensure selective and durable binding (Parker and Pratt, 2020; Fang et al., 2021) (**Figure 2**). The primary advantage of affinity probes lies in their ability to selectively target and capture active enzymes within native proteomes, bypassing the need for extensive purification or genetic manipulation. However, challenges such as non-specific binding and the complexity of probe design can limit their effectiveness (Tabana et al., 2023). Affinity probes have proven invaluable for mapping biosynthetic pathways in plants, as shown in studies on enzymes synthesizing steviol glycosides and other complex natural products.

## 3 UDP-glycosyltransferases in steviol glycosides biosynthesis

Steviol glycosides, the sweetening agents derived from *Stevia rebaudiana*, have been widely studied due to their potential as non-caloric sweeteners (Masand et al., 2024). Recent research utilizing a chemoproteomics-based strategy successfully identified the UDP-glycosyltransferases (UGTs) *Sr*UGT73E1, *At*UGT73C1 and *At*UGT73C5, which play a pivotal role in catalyzing the glycosylation of steviol to form steviol glycosides (Li et al., 2018; Zhou et al., 2018; Wong et al., 2020). The use of a photoaffinity probe specific to steviol, combined with mass spectrometry, allowed researchers to selectively profile the UGTs responsible for the final glycosylation steps. This discovery not only advances our understanding of the biosynthetic pathway of steviol glycosides but also offers a novel platform for the rapid identification of other enzymes involved in glycosylation, enabling synthetic biology approaches for scalable production.

## 4 Chalcomoracin biosynthesis through FAD-dependent cycloaddition

Chalcomoracin, a bioactive flavonoid isolated from mulberry (*Morus alba*), is synthesized through a highly unique flavin adenine dinucleotide (FAD)-dependent intermolecular Diels-Alder reaction. For years, the enzyme responsible for this cycloaddition reaction was unknown, despite its importance in the formation of chalcomoracin’s characteristic cyclohexene ring. Recent studies have identified a novel enzyme, *Morus alba* Diels–Alderase (*Ma*DA), through a biosynthetic intermediate probe (BIP)-based chemoproteomics strategy (Gao et al., 2020). *Ma*DA catalyzes the [4 + 2] cycloaddition with high specificity and enantioselectivity, marking the first discovery of a stand-alone intermolecular Diels-Alderase in plants (Gao et al., 2020, 2024). The use of chemoproteomics in this context allowed the functional characterization of this enzyme, which had remained inaccessible through traditional genomics or transcriptomics due to the lack of gene clustering in plant biosynthetic pathways.

## 5 Camptothecin biosynthesis and the role of OpCYP716E111

Camptothecin, an alkaloid with potent anti-cancer properties, is derived from *Camptotheca acuminata* and *Ophiorrhiza pumila* (Yang et al., 2021). The biosynthesis of camptothecin has long been a subject of study, with a significant gap in understanding the steps following strictosamide formation. A breakthrough came with the discovery of *Op*CYP716E111, an epoxidase responsible for catalyzing the conversion of strictosamide to strictosamide epoxide. Using a chemoproteomic approach, researchers designed a diazirine-based probe specific to strictosamide, which enabled the selective identification of *Op*CYP716E111 in the proteome of *Ophiorrhiza pumila* (Zhang et al., 2024). This discovery fills a critical gap in the camptothecin biosynthesis pathway and underscores the power of chemoproteomics to uncover previously unknown enzymes involved in complex plant metabolic processes.

## 6 The broader impact of chemoproteomics in plant biosynthesis

The discoveries surrounding steviol glycosides, chalcomoracin, and camptothecin highlight the broad applicability of chemoproteomics in the field of plant natural product biosynthesis (Li et al., 2018; Zhou et al., 2018; Gao et al., 2020; Wong et al., 2020; Zhang et al., 2024). Traditional methods such as transcriptomics and gene knockout studies often fall short in plants due to the dispersed nature of biosynthetic genes, making it difficult to pinpoint the enzymes responsible for each step. Chemoproteomics circumvents this issue by directly targeting enzyme activity through small molecule probes, allowing for rapid functional annotation of enzymes even in non-model plants. This approach is particularly advantageous in plants where secondary metabolism genes are not organized into clusters, a feature common in microbial systems but rare in plants. Chemoproteomics offers unique advantages for studying biosynthetic pathways, such as high sensitivity for detecting low-abundance enzymes without needing gene cloning or protein expression steps. It can also distinguish between closely related isoforms and profile multiple enzymes simultaneously, enabling a comprehensive view of metabolic networks in plant systems. These features make chemoproteomics particularly valuable for advancing research in natural product biosynthesis. Furthermore, the integration of chemoproteomics with synthetic biology holds the promise of sustainable production of these valuable compounds. By identifying and characterizing the enzymes involved in natural product biosynthesis, researchers can reconstitute these pathways in microbial hosts, enabling the scalable and controlled production of complex plant-derived compounds (Zhang et al., 2022; Gao et al., 2023).

Despite its advantages, chemoproteomics faces challenges in studying complex plant biosynthetic pathways. These include non-specific binding of probes, the need for extensive optimization in non-model plants, and reliance on high-quality mass spectrometry data for accurate enzyme identification. Additionally, challenges arise from probe design limitations that may affect binding efficiency and specificity. Future advancements, such as improved probe selectivity and integration with other omics technologies, hold promise for overcoming these obstacles and enhancing the approach’s utility in biosynthetic research.

## 7 Conclusion

Chemoproteomics has proven to be an indispensable tool in elucidating the biosynthetic pathways of complex natural products like steviol glycosides, chalcomoracin, and camptothecin. The ability to directly profile active enzymes involved in these pathways offers a new frontier in plant natural product research, accelerating the discovery of key biosynthetic genes and facilitating their application in synthetic biology. As this field continues to grow, chemoproteomics will likely play a central role in unlocking the full potential of plant-derived natural products for pharmaceutical and industrial applications.
